# Migration Behavior of ^90^Sr in the Soil–Plant System and Phytoremediation: A Review

**DOI:** 10.3390/plants15142208

**Published:** 2026-07-20

**Authors:** Yaowen Han, Xinyan Qiao, Han Yuan, Shaofei Cao

**Affiliations:** 1 China Institute for Radiation Protection, Taiyuan 030006, China; hanyaowen@cirp.org.cn (Y.H.);; 2 Key Laboratory of Radiation Environment & Health of the Ministry of Ecology and Environment, Taiyuan 030006, China; 3 CNNC Key Laboratory for Radiation Protection Technology, Taiyuan 030006, China

**Keywords:** ^90^Sr, radioecology, soil–plant system, phytoremediation

## Abstract

^90^Sr is a representative anthropogenic radionuclide, widely released into the environment through atmospheric nuclear tests, nuclear accidents, and routine operations of nuclear facilities, resulting in long-term residual contamination in soils worldwide. Its long half-life, high mobility, and chemical similarity to calcium make it easy to enter the food chain through the soil–plant system, thereby posing a persistent threat to ecosystems and human health. Conventional physical and chemical remediation approaches are often costly, ecologically disruptive, and inefficient for large-scale applications, highlighting an urgent need for sustainable, in situ strategies. Moreover, existing knowledge on ^90^Sr behavior has largely been generated from isolated studies, lacking an integrated framework to guide remediation efforts. This review summarizes the migration mechanisms of ^90^Sr in the soil–plant system and the main factors influencing its transport and accumulation. In soil, the migration of ^90^Sr is jointly controlled by soil texture and mineral composition, competing cations, organic matter, soil pH, and moisture, with cation exchange acting as the main immobilization mechanism. Plant uptake and accumulation of ^90^Sr show distinct inter- and intra-species differences, and the distribution generally follows the pattern of vegetative organs > reproductive organs. This process is regulated by root activity, transpiration, and competition with Ca^2+^ transport channels. Agronomic practices such as liming, deep plowing, and balanced fertilization can effectively reduce the phytoavailability of ^90^Sr by promoting ion competition and modifying the rhizosphere environment. Meanwhile, phytoremediation offers a promising green approach for the remediation of contaminated soils. Overall, this review provides a theoretical basis and scientific reference for the risk management and bioremediation of ^90^Sr in soil–plant systems.

## 1. Introduction

Atmospheric nuclear weapon tests conducted during the 20th century released large amounts of radioactive materials, causing global atmospheric deposition and a substantial increase in environmental radiation background levels [1]. These events left a profound and long-lasting impact on natural ecosystems and human living environments. In the short term, radioactive fallout resulted in severe local contamination; over time, however, it was transported worldwide through atmospheric circulation, leading to radionuclide accumulation in both densely populated regions and remote pristine areas [2]. Moreover, the large-scale development of nuclear energy has been accompanied by a series of severe nuclear accidents, most notably the Chernobyl and Fukushima disasters, which have further intensified the release and dispersion of radioactive substances into the atmosphere, soil, and water [3,4]. As a result, the remediation of nuclear contamination and the associated health risks have become urgent global concerns.

^90^Sr is a typical anthropogenic radionuclide and a pure β-emitter, produced mainly by the fission of ^235^U and ^239^Pu [5]. Owing to its relatively long physical half-life (28.9 years), high water solubility, strong mobility, and considerable fission yield, ^90^Sr is among the most important long-lived radioactive contaminants released by nuclear accidents and weapons tests, and it remains widely distributed in environmental media [6]. In addition, the ionic radius of Sr^2+^ (2.00 Å) is close to that of Ca^2+^ (1.80 Å) [7], which results in similar geochemical and physiological behaviors. Consequently, ^90^Sr is readily mistaken for calcium by living organisms and can be taken up and transferred through biological systems, accumulating in plants, animals, and ultimately humans. This process may enhance its transfer along food chains and intensify environmental risks. The International Agency for Research on Cancer (IARC) has classified ^90^Sr as a Group 1 human carcinogen in its latest monographs [8]. After entering the body, ^90^Sr preferentially accumulates in calcium-rich tissues such as bones and teeth. The high-energy β-particles emitted during its decay can damage cellular structures and DNA, thereby increasing the risk of cancers such as bone cancer and leukemia [9], and posing a serious threat to human health.

**Figure 1:**
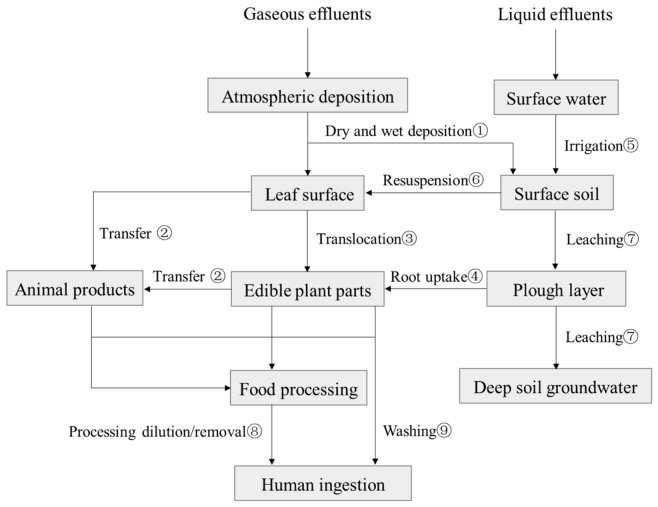
Transfer of radionuclides to humans via the soil–plant system (1—dry and wet deposition of radionuclides; 2—transfer from plants to animal products; 3—translocation to edible plant parts; 4—root uptake, transfer from soil to plants; 5—irrigation; 6—soil resuspension; 7—migration in soil; 8, 9—human ingestion after food processing and washing).

Over the past several decades, the environmental behavior of ^90^Sr, particularly its migration in the soil–plant system, has remained a major focus of radioecological research. Soil is the main sink for radionuclides entering terrestrial environments and serves as a key interface for their transport and transformation. The sorption, ion exchange, and fixation of ^90^Sr in soil not only determine its movement within the soil profile, but also directly influence its bioavailability in ecosystems [10]. As the primary link between soil and the food chain, plants absorb Sr^2+^ from soil solution through their roots and translocate it to aboveground tissues, including stems, leaves, and even edible parts, via the transpiration stream and xylem transport [11]. In this way, ^90^Sr is transferred stepwise through trophic levels, creating a direct risk to food safety (Figure 1). Radioactive contamination surveys have shown a direct correlation between ^90^Sr activity concentrations in red canary grass (*Phalaris arundinacea*) growing near a nuclear reactor and those detected in the eggshells of Canada geese feeding on that grass [12]. Field investigations in the Chornobyl-contaminated Ivankiv district further demonstrated substantial transfer of ^90^Sr from soil to culinary grains and forest products, highlighting the continued importance of the soil–plant pathway as a source of human dietary exposure [13]. At the same time, the uptake and transport of ^90^Sr by plants are strongly affected by species identity, growth stage, root activity, and rhizosphere conditions, showing marked biological variability [14].

Existing knowledge on ^90^Sr behavior in the soil–plant system has largely been generated through studies targeting isolated aspects, such as plant uptake, transfer parameters, or remediation techniques. However, these processes have rarely been integrated into a comprehensive framework describing ^90^Sr migration through the soil–plant system. Therefore, this review systematically summarizes ^90^Sr migration behavior in soil–plant systems, evaluates the key factors controlling its mobility and bioavailability, and critically discusses current agronomic and phytoremediation strategies, aiming to provide an integrated framework for risk assessment and sustainable remediation of ^90^Sr-contaminated environments.

## 2. Sources of ^90^Sr in Soil

^90^Sr enters terrestrial environments through a variety of anthropogenic activities, including atmospheric nuclear weapon tests, nuclear accidents, routine operation of nuclear facilities, and industrial and medical applications. These sources differ substantially in their release characteristics, spatial distribution patterns, and environmental persistence. The major sources of ^90^Sr in soil and their main characteristics are summarized in Table 1.

### 2.1. Nuclear Activities and Accidents

#### 2.1.1. Atmospheric Nuclear Weapon Tests

The atmospheric nuclear weapon tests conducted in the mid-20th century represent the most important historical source of ^90^Sr on a global scale [15]. These tests released vast quantities of anthropogenic radionuclides, including ^90^Sr, ^137^Cs and ^131^I. The total explosive yield of the atmospheric tests amounted to approximately 428 megatons, equivalent to roughly 29,000 Hiroshima-sized bombs [16,17]. The radioactive materials produced were dispersed worldwide through atmospheric circulation, forming what is commonly called “fallout”, and eventually deposited on the Earth’s surface [18]. Following atmospheric nuclear detonations, ^90^Sr was incorporated into radioactive fallout particles and deposited onto vegetation and soils [19]. After deposition, weathering and dissolution gradually released Sr^2+^ into the soil solution, making it available for plant uptake. Although most atmospheric testing ceased after the signing of the Partial Test Ban Treaty in 1963, the ^90^Sr and other radionuclides deposited in the preceding years continue to cycle in the environment and can still be detected in soil and biological samples worldwide [1,20].

#### 2.1.2. Nuclear Accidents

The 1986 Chernobyl accident is acknowledged as the most severe nuclear accident, given the enormous release of radionuclides and its far-reaching impact. The accident generated a radioactive plume that traveled long distances, leaving characteristic contaminants in many European countries and even beyond [21]. The ^90^Sr released during the Chernobyl and Fukushima accidents was largely associated with nuclear fuel particles. These particles display relatively weak atmospheric mobility, and consequently the deposition of ^90^Sr exhibited a strongly localized pattern, concentrating in areas close to the accident sites. For example, within the 30 km exclusion zone around the Chernobyl site, the ^90^Sr deposition in soils was markedly higher than in the outer regions [22]. Shortly after the Fukushima accident, elevated ^90^Sr concentrations (up to 1000 Bq/kg) and ^137^Cs concentrations (up to 4600 kBq/kg) were also detected in surface soils near the Fukushima Daiichi Nuclear Power Plant (NPP) [23]. Notably, these ^90^Sr-bearing fuel particles undergo prolonged weathering under ambient environmental conditions, gradually transforming into soluble forms. By enhancing their mobility and bioavailability, this process gradually poses a long-term secondary risk to ecosystems and public health [24].

#### 2.1.3. Routine Operation of Nuclear Facilities

The routine operation of nuclear facilities serves as a persistent source of ^90^Sr and other radionuclides [25]. During nuclear power generation, ^90^Sr is produced as a uranium fission product; although releases are tightly regulated, chronic low-level discharges through liquid and gaseous effluents can still occur, leading to gradual accumulation in environmental media [26]. Spent fuel reprocessing plants represent another potentially significant source [27]. During spent fuel reprocessing, ^90^Sr is separated from irradiated fuel into high-level liquid waste streams through chemical separation processes [28]. Although radionuclide releases from reprocessing facilities are strictly regulated and generally remain at very low levels, their long-term environmental fate and potential ecological impacts continue to be important subjects of environmental monitoring and radiological risk assessment.

### 2.2. Other Industrial and Technological Applications

Beyond nuclear power production, the use of ^90^Sr in industrial and medical applications also represents a noteworthy pathway for its release into the environment. Radioisotope Thermoelectric Generators (RTGs) have been widely employed to convert the decay heat of ^90^Sr into long-term, stable electrical power for remote meteorological stations, maritime navigational aids, and deep-space probes [29]. Although these devices incorporate multiple containment barriers designed to confine the radioactive material under extreme conditions, ^90^Sr leakage can still occur during long-term field deployment or decommissioning as a result of physical damage, corrosion, or management lapses, leading to localized environmental contamination [30,31].

In medicine, ^90^Sr has been utilized as a pure β^−^ source in brachytherapy, particularly for the treatment of superficial tumors and specific ophthalmic disorders such as pterygium [32,33,34,35]. The emitted β-particles have a short range and can be precisely targeted, thereby minimizing damage to surrounding healthy tissues [33]. However, inadequate control of medical ^90^Sr sources during their life cycle may lead to localized releases and environmental contamination. Although such contributions are minor compared with those from nuclear weapons testing, reactor accidents, and fuel-cycle activities, they remain relevant for environmental surveillance and radiation protection.

## 3. Environmental Behavior of ^90^Sr in Soil

### 3.1. Distribution Patterns of ^90^Sr in Soil

The average Sr content in soil is approximately 0.035%, which is close to its Clarke value in the Earth’s crust [36], although values as high as 0.2% have been reported in some regions [12]. Sr is moderately mobile in soils and sediments, largely due to sorption by soil constituents such as clay minerals and organic matter [37]. In soil, ^90^Sr is generally present as water-soluble complexes or free Sr^2+^, and its environmental chemistry is dominated by the divalent cation form. Its vertical distribution is strongly influenced by soil structure and moisture conditions. In most cases, ^90^Sr concentrations are highest in the surface layer, particularly within the top 0–10 cm, and decrease progressively with depth, reflecting both retention by the solid phase and downward redistribution driven by percolating water [38,39]. Studies show that ^90^Sr migrates downward more rapidly than other common radionuclides such as ^137^Cs, ^241^Am, and ^239/240^Pu across a range of soil types, from French mineral sandy soils and organic soils [40] to alpine pasture soils in Austria and Switzerland [41,42]. Annual downward migration rates of ^90^Sr in soil have been reported to be 0.7–1.5 cm [43], yet the bulk of residual ^90^Sr remains concentrated in the surface layer, making this zone the most vulnerable to radioactive contamination.

### 3.2. Factors Affecting ^90^Sr Migration in Soil

The migration of ^90^Sr in soil is controlled by multiple factors, including soil texture and mineral composition, competing cations, organic matter content, moisture conditions, and pH. These factors act through cation exchange, surface complexation with organic matter, precipitation–dissolution reactions, and the formation of soluble organic complexes. Among these processes, cation exchange is generally considered the dominant mechanism [44].

#### 3.2.1. Soil Texture and Mineral Composition

Soil texture and mineral composition fundamentally determine the effective capacity of the cation exchange system. Sr^2+^ is reversibly adsorbed onto permanent negatively charged sites of clay minerals primarily through electrostatic attraction, forming outer-sphere complexes [45]. Soil sorption sites can be grouped into three categories according to their selectivity: regular exchange sites (RES) on the planar surfaces of clay particles, frayed edge sites (FES) at the edges of phyllosilicate minerals, and highly specific high-affinity sites (HAS). For ^90^Sr, sorption occurs mainly on RES through a simple and reversible ion-exchange process [10,46]. Mineral composition, particularly the type and content of clay minerals, is the main factor controlling the fixation and mobility of ^90^Sr. 2:1-type clay minerals such as montmorillonite and vermiculite, with their large specific surface area and high cation exchange capacity (CEC), exhibit very strong adsorption (up to 92–99.9%). In contrast, 1:1-type clay minerals like kaolinite show weaker adsorption (40–68%), while primary minerals such as quartz and feldspars show the lowest capacity (10–50%) [10]. Adsorption experiments demonstrated that a higher soil CEC enhances the adsorption of Sr through ion-exchange processes [47]. Consequently, fine-textured soils rich in secondary clay minerals such as montmorillonite strongly immobilize ^90^Sr, whereas coarse-textured sandy soils with low CEC and limited clay content favor its migration. Dynamic column experiments have shown that the peak migration distance of ^90^Sr is greatest in sandy soil, intermediate in silty loam, and smallest in clay soil [48]. In highly weathered acidic soils such as Brazilian Ferralsols, more than 80% of ^90^Sr remained in a potentially mobile form two years after contamination [49].

#### 3.2.2. Competing Cations

The migration of ^90^Sr in soil is also controlled by competition from other cations for sorption sites and root uptake pathways. In general, the competitive strength follows the order Al^3+^ > Fe^3+^ > Ba^2+^ > Ca^2+^ > Mg^2+^ > K^+^ > NH_4_^+^ > Na^+^, and higher concentrations of competing cations usually increase the mobility of ^90^Sr. Among these cations, Ca^2+^ is particularly important because it has the same charge and a similar ionic radius and coordination behavior [45]. As a result, elevated Ca^2+^ concentrations in the soil solution can strongly suppress ^90^Sr sorption and enhance its activity and mobility. The content of exchangeable Ca in soil has been identified as a key factor determining the bioavailability of ^90^Sr [50,51]. In soils containing Ca-bearing minerals such as calcite and dolomite, ^90^Sr can also be immobilized through isomorphic substitution, in which Sr^2+^ replaces Ca^2+^ in the crystal lattice, or through co-precipitation [10]. In addition, different cations can compete for surface hydroxyl (≡SOH) sites on oxide surfaces, thereby altering surface complexation reactions and affecting Sr adsorption [52].

#### 3.2.3. Soil Organic Matter

Soil organic matter exerts a dual effect on the environmental behavior of ^90^Sr, promoting both immobilization and mobilization. In general, plant uptake of ^90^Sr decreases as soil organic matter increases [53], because functional groups such as carboxyl and phenolic hydroxyl groups on humic substances can bind Sr^2+^ through surface complexation. Moreover, humification enhances soil CEC and facilitates the formation of organo-mineral complexes, which further stabilizes retained ^90^Sr [54]. A study examined ^90^Sr sorption in peaty-podzolic gley soil and alluvial meadow gley soil and found that, in organic-rich horizons, ^90^Sr was mainly fixed by complexation with organic matter, whereas ion exchange dominated in mineral and organo-mineral layers [55]. On the other hand, Sr^2+^ also displays a strong affinity for low-molecular-weight soluble organic ligands such as fulvic acid. Fulvic acid fractions and low-molecular-weight organic acids generated by microbial decomposition can form dissolved complexes with Sr^2+^, thereby increasing its mobility [56]. The overall effect of organic matter is therefore not uniform, but rather reflects a dynamic balance among fixation, mobilization, and redistribution.

#### 3.2.4. Soil Moisture

Under sufficient moisture, ^90^Sr exists mainly in dissolved forms, including free Sr^2+^ and soluble organic–Sr complexes, which facilitates mass flow and diffusion toward the rhizosphere and enhances uptake by roots through Ca analogue pathways, resulting in elevated bioavailability [49]. Soil column experiments have shown that larger leaching volumes drive the ^90^Sr concentration peak to deeper layers [57], indicating that heavy rainfall or excessive irrigation may transport dissolved ^90^Sr downward with percolating water, potentially moving it below the root zone and even toward groundwater. Under dry conditions, by contrast, liquid-phase transport is limited, and ^90^Sr is more readily adsorbed or fixed onto particle surfaces, which reduces bioavailability. However, the influence of moisture is not strictly linear. Studies indicate that when the soil becomes water-saturated, the mobility of ^90^Sr may actually decrease [58]. Compared with field-capacity conditions, saturation leads to a rapid decline in redox potential, triggering biogeochemical processes such as the reductive dissolution of Fe/Mn oxides and the formation of carbonate minerals, which can effectively decrease the soluble and exchangeable fractions of ^90^Sr and thus reduce its activity [59,60].

#### 3.2.5. Soil pH

Soil pH is a critical chemical factor regulating the environmental behavior and phytoavailability of ^90^Sr. In general, ^90^Sr bioavailability decreases as soil pH increases and increases as pH decreases [61]. Raising soil pH from 4.5 to 7.4 has been shown to reduce the transfer factor (TF) of ^90^Sr by a factor of 1.7 [62]. The lower availability in alkaline soils can be explained by two main mechanisms. First, higher pH increases the negative charge on variable-charge surfaces of soil colloids, thereby strengthening electrostatic adsorption of Sr^2+^ [63]. Second, ^90^Sr may form sparingly soluble precipitates such as SrCO_3_ with carbonate ions or be co-precipitated with Ca in carbonate minerals [61]. In acidic soils, by contrast, H^+^ competes for sorption sites and displaces other cations, releasing previously adsorbed ^90^Sr together with nutrient cations such as Ca^2+^ and K^+^ into the soil solution and enhancing their availability for root uptake [55]. In addition, pH can alter the solubility of soil organic matter and Fe/Mn oxides, thereby affecting the complexation and sorption behavior of ^90^Sr [64].

## 4. Uptake and Transport of ^90^Sr by Plants

### 4.1. Mechanisms of ^90^Sr Uptake and Accumulation from Soil

Several transfer parameters have been proposed to quantify the ability of plants to accumulate radionuclides from soil. Among them, the soil-to-plant transfer factor (TF, also referred to as F_v_) is the most widely used, whereas the concentration ratio (CR) and aggregated transfer factor (T_ag_) are applied less frequently. TF (or F_v_) is defined as the activity concentration of a radionuclide in plant tissue (Bq/kg, dry weight) divided by its activity concentration in soil (Bq/kg, dry weight) [65,66]. CR is defined as the activity concentration in an organism (Bq/kg, fresh weight) divided by that in the environmental medium (for soil: Bq/kg, dry weight) [67]. T_ag_ is defined as the activity concentration in plants (Bq/kg, dry weight) divided by the total inventory per unit area of soil (Bq/m^2^) [68]. Some authors argued that T_ag_ may reduce the bias introduced by the vertical distribution of radionuclides in soil profiles when comparing different datasets [68,69]. These transfer parameters have been widely incorporated into food-chain transport models as integrative indicators for predicting radiation exposure risks associated with the entry of ^90^Sr into the human diet via crops [70]. However, applying such empirical parameters as if they were universal constants for estimating plant activity concentrations requires considerable caution. Long-term observations have shown that the TF values of many long-lived radionuclides can vary by more than three orders of magnitude [71]. Even for a specific soil-crop system, TF values for radiocesium may differ by as much as three orders of magnitude [72]. Such large variability reflects the fact that TF is an integrated parameter influenced by multiple processes, including soil chemistry, soil biology, hydrology, and plant physiology, all of which are themselves highly variable and further affected by climate and agricultural management.

**Figure 2:**
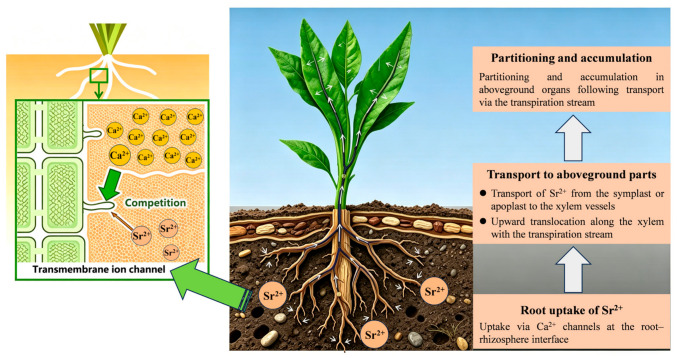
Mechanism of Sr uptake by plants [6,38].

The root system is the main pathway through which plants take up ^90^Sr from soil, and root uptake far exceeds foliar absorption [11]. The uptake of Sr^2+^ can generally be divided into three steps: (1) root absorption, during which Sr^2+^ enters the symplast through Ca^2+^ transport channels in the root-cell plasma membrane; (2) radial transport and xylem loading, during which Sr^2+^ moves through the symplastic and/or apoplastic pathways to the xylem and is then transported upward by the transpiration stream [73]; and (3) accumulation in plant organs, during which Sr^2+^ is transferred from xylem vessels into adjacent parenchyma tissues, such as mesophyll and fruit flesh (Figure 2). Once Sr^2+^ has been delivered to a given organ through transpiration, its further redistribution is usually limited. Because ^90^Sr behaves chemically in a manner similar to Ca, their xylem transport patterns are also similar [74]. As a result, plant organs with higher transpiration rates generally accumulate more ^90^Sr. In addition, xylem structure, including vessel diameter and connectivity, can also influence transport efficiency [75]. Root metabolic activity further affects ^90^Sr uptake, since organic acids and other exudates released by roots may alter the rhizosphere pH, thereby increasing the solubility and bioavailability of ^90^Sr and modulating its absorption [76].

### 4.2. Factors Influencing Plant Uptake of ^90^Sr from Soil

#### 4.2.1. Phylogenetic Characteristics

The ability of plants to accumulate ^90^Sr depends strongly on their phylogenetic position and inherent biological traits, with marked differences at the levels of family, genus, species, and even cultivar. In general, dicotyledonous crops tend to accumulate more Sr than monocotyledonous crops. This pattern may be related to the higher cation exchange capacity of dicot roots and is further influenced by soil Ca availability, plant species, and environmental conditions [77]. Leafy vegetables, such as cabbage and spinach, generally exhibit relatively high Sr accumulation capacities compared with other crop groups [70]. Evidence from Kuwaiti arid soils indicates that the soil-to-plant transfer of Sr and Ba is highest in leafy vegetables, followed by root crops and non-leafy vegetables [78]. However, radionuclide accumulation is only weakly associated with broad taxonomic groups and is mainly determined by species-specific traits [79]. Even within the same family or genus, considerable differences in accumulation capacity may occur among species [80,81,82]. For example, a study at the East Ural Radioactive Trace (EURT) site showed that plant species accounted for about 55% of the variation in ^90^Sr accumulation [83]. Similarly, data from underground nuclear explosion craters at the Semipalatinsk Test Site indicated that TF values varied by approximately 5–6 times among different vegetation types [84].

At the intraspecific level, a clear genetic basis for ^90^Sr accumulation has been demonstrated in crops. For example, significant cultivar-dependent variation in Sr accumulation and translocation capacities was observed among nine sunflower (*Helianthus annuus* L.) cultivars grown in Sr-contaminated soil [85]. Up to 2.6-fold variation in ^90^Sr uptake has been reported among 28 winter wheat cultivars [86], and significant genotypic differences in grain Sr content have been observed across diverse wheat genotypes (AA, BB, BBAA, BBAADD, DD) [87]. The selection of low-accumulating genotypes has accordingly been proposed as a practical strategy to improve both food safety and product quality. These findings collectively indicate that the substantial variation in Sr accumulation, both within and among species, serves as a valuable basis for screening crop cultivars with low accumulation potential. Therefore, for the safe agricultural use of radiologically contaminated soils, such genetic differences should be fully taken into account, and low-accumulation varieties should be preferentially selected in order to reduce the risk of ^90^Sr entering the food chain.

#### 4.2.2. Growth and Developmental Stage

Plant uptake and accumulation of ^90^Sr are also significantly influenced by growth stage [88]. It has been observed that TF values change with crop development, typically being lower at harvest than during the middle of the growing season, a pattern attributed to declining physiological activity near maturity and the dilution effect from increasing biomass [88]. Experiments in which ^90^Sr contamination was introduced at different growth stages of lettuce and winter wheat have demonstrated that TF values are highest after early exposure, such as at germination, and decline progressively as contamination is delayed [89]. During the seedling stage, the root system is still developing but exhibits strong absorption capacity and efficient upward transport, making plants particularly vulnerable [90]. At the mid-growth stage, root systems expand and penetrate deeper soil layers, increasing the absorptive area and stabilizing uptake [61]. By maturity, uptake generally declines, and ^90^Sr tends to accumulate mainly in non-edible tissues such as roots and stems, whereas concentrations in grains or fruits are usually low [91].

#### 4.2.3. Soil Type

The accumulation of radionuclides by plants is closely linked to soil type. In general, ^90^Sr uptake is greater in light-textured, infertile, acidic soils with low Ca saturation, whereas uptake is strongly suppressed in fertile, Ca-rich soils such as chernozems because of competition from Ca^2+^ [49,50,53]. A clear negative relationship between ^90^Sr transfer from soil to grass and soil Ca concentration has been demonstrated, with lower Ca contents corresponding to higher TF values, and vice versa [92].

The influence of soil type on ^90^Sr transfer is largely governed by differences in soil physicochemical properties. Soils with higher clay mineral content, CEC, and exchangeable Ca generally exhibit stronger ^90^Sr retention and lower soil-to-plant transfer than coarse-textured or highly weathered soils [55]. Consistent with this mechanism, TF values for both ^137^Cs and ^90^Sr in Entisols were reported to be 1.03–4.86 times higher than those in Inceptisols in semi-arid Syrian soils [88]. This difference has been attributed to the lower pH, lower clay content, and lower exchangeable Ca and K concentrations, but higher organic matter content, in Entisols, all of which reduced the soil’s retention capacity and increased radionuclide bioavailability. Similar patterns have been reported for other soils. For instance, highly weathered ferralitic soils generally yield higher TF values than chernozems [49]. Chernozems contain abundant 2:1-type clay minerals such as vermiculite, which provide high cation exchange capacity through permanent negative charges and thereby effectively adsorb and immobilize cations such as ^90^Sr. By contrast, ferralitic soils are deeply weathered and their clay fraction is dominated by low-activity 1:1-type minerals such as kaolinite, which have low CEC [93]. Their typically acidic pH further weakens specific cation adsorption, leaving ^90^Sr more mobile in soil solution and therefore more available for plant uptake.

### 4.3. Distribution and Translocation of ^90^Sr Within Plants

The distribution and translocation of ^90^Sr within plants show marked organ-specific differences, with a behavioral pattern closely resembling that of its chemical analogue Ca. ^90^Sr tends to accumulate preferentially in lignified tissues and senescing leaves [94]. In most cases, concentrations in vegetative organs such as stems and leaves are far higher than those in reproductive organs, including seeds and fruits, with differences ranging from one to four orders of magnitude [90]. This pattern is largely due to the limited xylem connectivity and weak transpiration stream in developing fruits, which restricts the entry of ^90^Sr. A substantial body of evidence supports this general trend. For instance, Sr^2+^ was found to accumulate mainly in soybean stems, which contained 87.4–90.4% of the total plant ion burden [95]. In apricot, grapevine, and olive trees, ^90^Sr TF values were highest in leaves and lowest in fruits, olive oil, and apricot kernels [96]. In the semi-arid regions of Turkey, the ^90^Sr concentrations in the grains of barley, beans, and apples ranged from 0.13 to 0.32 Bq/kg, while those in the stems ranged from 0.10 to 2.57 Bq/kg [65]. Similar patterns have been reported in clover, cereals such as wheat, oat, barley and rice, legumes such as broad bean and sesame, and silver birch, where ^90^Sr concentrations were highest in leaves or shoots and lowest in grains or trunks [66,88,97,98,99,100,101]. Grain concentrations were consistently and substantially lower than those in vegetative tissues. Nevertheless, exceptions do exist, indicating that distribution patterns are influenced by both plant species and growing conditions. For example, in hemp, Sr has been reported to be mainly distributed in roots (45%) and stems (40%), with the lowest proportion in leaves (15%) [102].

### 4.4. Phytotoxicity and Growth Effects of ^90^Sr

Although Sr is abundant in the Earth’s crust, it is not an essential element for plant nutrition and can become toxic at elevated concentrations. Plants readily absorb Sr through the Ca transport system, and excess Sr can interfere with a range of cellular processes by competing with and displacing Ca^2+^, effectively inducing Ca deficiency [103]. Previous studies have documented the negative effects of excess Sr on plant growth and development, including inhibition of root elongation, reduction in biomass, decrease in chlorophyll and carotenoid contents, and impairment of photosynthetic efficiency [95,104,105,106,107,108]. Higher Sr concentrations were associated with alterations in leaf anatomical parameters in plants growing under contaminated conditions [109]. Sr toxicity can also trigger oxidative stress through reactive oxygen species (ROS) accumulation, activation of antioxidant enzymes, and increased production of secondary metabolites [105,110,111]. In addition, Sr stress may disturb miRNA biosynthesis [107]. With increasing external Sr concentration and exposure duration, the photosynthetic oxygen evolution rate, chlorophyll content, and the activities of ribulose-1,5-bisphosphate carboxylase/oxygenase (Rubisco) and phosphoenolpyruvate carboxylase (PEPCase) in oilseed rape leaves all showed progressive declines [104]. These findings indicate that Sr accumulated in leaves impairs multiple processes of photosynthesis, including light energy absorption, energy transfer, and photosynthetic carbon assimilation. In *Arabidopsis thaliana*, both low and high Sr concentrations altered chlorophyll fluorescence, and concentrations above 0.01 mM significantly inhibited root and leaf dry weight [103]. Because plants cannot effectively discriminate between stable Sr and ^90^Sr, the latter is readily absorbed from soil and accumulated in plant tissues, posing a serious threat to ecosystems, agricultural productivity, and human health [14].

### 4.5. Influence of Agronomic Practices on Plant Uptake of ^90^Sr

When crop cultivar substitution is not feasible, appropriate agronomic management can serve as an effective complementary strategy to reduce ^90^Sr transfer. Fertilization alters the concentrations of major nutrient ions such as K^+^, Ca^2+^, and Mg^2+^ in the soil solution and thereby influences the bioavailability of Sr^2+^ in the soil–plant system, mainly through competitive ion adsorption [71,112]. Among the available measures, liming has proven to be one of the most effective ways to suppress Sr uptake by crops, particularly in acidic or Ca-deficient soils. In pot experiments, increasing soil Ca application has been shown to reduce Sr uptake in oilseed rape and oat by as much as 65% and 60%, respectively [77]. Organic amendments such as compost and biochar can also affect the bioavailability of Sr^2+^ by supplying competing nutrient ions and increasing the number of sorption sites [113]. Biochar and digestate, which are widely used by-products of bioenergy production, are increasingly being applied to agricultural soils; they can influence ^90^Sr availability by altering soil organic matter content, ionic strength, and pH [114]. Deep plowing is another important countermeasure. In natural pastures in Russia, Belarus, and Ukraine, agronomic measures including plowing combined with deep tillage, liming, and NPK fertilization have been reported to reduce the transfer of both ^137^Cs and ^90^Sr to plants. Among these treatments, the combination of plowing and deep tillage was the most effective, lowering ^90^Sr transfer to plants by a factor of 2–4 in the second year [115].

## 5. Application of Phytoremediation to ^90^Sr Radioactive Contamination

The expanding use of nuclear energy, together with the persistent risk of accidental releases from nuclear facilities, has made the development of efficient and sustainable remediation technologies for radiologically contaminated soils an urgent priority. Current remediation technologies can be broadly classified into physical, chemical, and biological approaches, each with distinct advantages and limitations (Table 2) [85,116,117]. Among these, biological remediation has attracted increasing attention because of its low cost, minimal environmental disturbance, and potential for in situ application.

Phytoremediation is a green remediation technology that employs plants and their associated microbial communities to remove, transform, or immobilize pollutants in the environment [118,119]. Plants absorb radionuclides from soil through their roots and translocate them to aboveground parts for accumulation, a process that is influenced by plant species, soil physicochemical properties, and environmental conditions. This approach relies primarily on plant metabolic activity and synergistic interactions with rhizosphere microorganisms, and can achieve contaminant removal or stabilization through phytoextraction, phytovolatilization, phytostabilization, and microbial degradation [120]. Owing to its relatively low cost and limited ecological disturbance, phytoremediation has attracted increasing attention as a promising remediation strategy, particularly in areas with low to moderate contamination levels in soil [116,121,122].

### 5.1. Screening of Plants for Phytoremediation

Screening suitable plant species is a critical step in determining the success of phytoremediation. Ideal candidate plants should combine high tolerance to ^90^Sr, strong bioconcentration capacity, and substantial biomass production. A number of species with potential for ^90^Sr phytoremediation have been identified. Table 3 lists several plants reported for Sr remediation, including *Vetiveria zizanioides*, *Euphorbia macroclada*, *Salvia verbenaca*, *Alyssum murale*, *Broussonetia papyrifera*, *Parthenocissus tricuspidata*, and *Avena sativa*, among others. In recent years, researchers have proposed using agricultural crops as an alternative for cleaning up contaminated soils, arguing that the high biomass and rapid growth of crops can compensate for their moderate accumulation capacity [123,124,125]. To date, Sr uptake and distribution have been investigated in various crops, including rice, wheat, barley, sunflower, and oat. In a study assessing the phytoremediation potential of 26 cultivars of wheat, hulled oat, naked oat, and barley, shoot Sr concentrations at the tillering stage were found to be significantly higher in naked oat and barley than in wheat (*p* < 0.05). Among them, the naked oat cultivar “Neimengkeyimai-1” exhibited the highest Sr concentration, suggesting it might be a promising candidate for decontaminating polluted soils [100]. In efforts to reduce radionuclide accumulation in crops, cultivar identity is as important as species identity. Previous studies have shown that ^90^Sr accumulation can differ by up to an order of magnitude among cultivars of the same species, such as potato [10]. It should also be noted that part of the absorbed ^90^Sr may be retained in non-economic biomass fractions, such as straw. If these residues are returned to the field by incorporation, ^90^Sr may be reintroduced into the soil, resulting in secondary contamination.

### 5.2. Plant–Microbe Combined Remediation Technology

The combined use of plants and microorganisms represents a sustainable strategy for remediating radioactively contaminated soils [121]. The rhizosphere harbors a diverse microbial community that contributes to soil fertility and plant growth through processes such as decomposition, mineralization, and the fixation of soil micronutrients [120]. Microorganisms can also mobilize radionuclides in soil by secreting organic acids and other substances, thereby modifying their chemical speciation and enhancing plant uptake and accumulation of Sr [12,52]. To improve remediation performance, a variety of plant–microbe combined systems have been developed for Sr-contaminated soils. In a pasture–microorganism combined remediation study, inoculation with three dominant microbial consortia (E, G, and H) was shown to significantly enhance Sr accumulation in Sudan grass and sorghum–Sudan grass hybrids, increasing uptake by 0.5- to 4-fold compared with the control [134]. Metagenomic analysis indicated that microbial inoculation raised the abundance of *Bacillus* in the rhizosphere, thereby improving disease resistance and stress tolerance in the grasses and ultimately enhancing remediation efficiency. Inoculation with the arbuscular mycorrhizal fungus *Glomus geosporum* has been found to promote Sr accumulation and tolerance in sorghum, with significantly higher Sr contents in leaves and roots compared to non-mycorrhizal plants (*p* < 0.05) [123].

### 5.3. Plant-Chelator Combined Remediation Technology

In recent years, plant-chelator combined remediation has emerged as a research hotspot and has been widely applied in practice. The plant-chelator combined remediation strategy is based on the principle that chelating agents convert relatively immobile Sr in soil into plant-available forms, after which hyperaccumulator plants absorb the element and translocate it to aboveground tissues for harvest removal [136]. For example, synthetic chelating agents such as ethylenediaminetetraacetic acid (EDTA) can mobilize heavy metals in soil, thereby enhancing their migration to roots and increasing plant extraction efficiency [137]. Treatments with EDTA, citric acid, and ascorbic acid have all been shown to increase Sr accumulation in tobacco relative to the control [138,139]. More recently, field trials in radionuclide-contaminated farmland showed that integrating high-^90^Sr-accumulating leafy vegetables with split low-dose citric acid applications (5 mmol/kg soil) increased total ^90^Sr phytoextraction by 62–78%, demonstrating the effectiveness of combining crop selection with biodegradable chelators under field conditions [70]. Given that EDTA is environmentally persistent and may pose environmental risks [140], future research should explore biodegradable chelators, such as natural organic acids including citric acid, malic acid, and tartaric acid, as well as novel synthetic ligands with improved environmental compatibility. Such efforts will be essential for balancing remediation efficiency with environmental safety.

## 6. Conclusions

### 6.1. Summary

(1) The migration of ^90^Sr in soil is governed by the combined effects of sorption on clay minerals (especially high-CEC minerals such as montmorillonite and vermiculite), competing cations, complexation with organic matter, soil pH, and soil moisture, with cation exchange being the primary immobilization mechanism. In acidic, sandy, and calcium-poor soils, ^90^Sr exhibits much higher mobility and phytoavailability, whereas in alkaline, clay-rich, and calcium-rich soils, its activity is effectively reduced through competitive sorption and precipitation.
(2) Plant uptake of ^90^Sr displays pronounced biological variability and organ specificity. The accumulation capacity is largely determined by genetic traits at the species and cultivar levels, and may differ by orders of magnitude among cultivars of the same crop. Within plants, ^90^Sr tends to accumulate preferentially in metabolically active or senescing vegetative organs such as leaves and stems, while reproductive organs, including grains and fruits, generally contain lower concentrations because of their limited xylem connectivity and weaker transpiration stream.
(3) Agronomic practices can serve as a crucial supplementary strategy for reducing ^90^Sr transfer to crops. Liming, mineral fertilization, deep plowing, and the incorporation of organic amendments can substantially suppress ^90^Sr uptake by promoting ion competition, increasing sorption capacity, or altering rhizosphere conditions. Meanwhile, phytoremediation strategies, including the selection of high-accumulating plant species, the construction of plant–microbe consortia, and the use of chelating agents, can enhance either the extraction or stabilization of ^90^Sr, offering a promising green remediation option for soils with low to moderate contamination.

### 6.2. Perspectives

Future work may focus on the following aspects to advance the understanding of ^90^Sr migration in the soil–plant system and to improve phytoremediation performance:

(1) Developing coupled dynamic prediction models that integrate soil–plant–microbial processes and key transfer parameters, enabling a shift from qualitative description to quantitative prediction and offering tools for precise risk assessment and management decisions under diverse environmental scenarios.
(2) Clarifying the molecular mechanisms underlying plant–microbe interactions, including how root exudates shape rhizosphere microbial communities and how microbial metabolites, such as organic acids, siderophores, and extracellular polymeric substances, influence the chemical speciation of ^90^Sr through dissolution, complexation, and immobilization.
(3) For phytoremediation, two directions deserve particular attention. First, plant–microbe consortia with high tolerance and strong ^90^Sr accumulation capacity should be screened or engineered, with gene-editing tools used where appropriate to improve the uptake and translocation traits of candidate plants. Second, the long-term remediation efficiency and ecological safety of different plant–microbe combinations should be evaluated under varying soil and climatic conditions, so as to facilitate the transition from laboratory studies to field-scale application.

## Figures and Tables

**Table 1:** Main sources and characteristics of ^90^Sr in soil.

Sources	Release Characteristics	Main Distribution Pattern	Environmental Persistence
Atmospheric nuclear tests	Global fallout	Relatively uniform distribution	Long-term (half-life 28.8 years)
Nuclear accidents	Regional contamination	Irregular patchy distribution	Long-term
Nuclear power plant operation	Local discharge	Gradient distribution around point source	Medium to long term
Industrial and medical uses	Point-source pollution	Local contamination	Depends on management practices

**Table 2:** Comparison of the major remediation strategies for radionuclide-contaminated soils.

Remediation Strategy	Representative Techniques	Advantages	Limitations
Physical remediation	Excavation; soil replacement; capping; vitrification; electrokinetic remediation	Rapid contaminant removal; suitable for heavily contaminated sites	High cost; severe soil disturbance; secondary radioactive waste; limited scalability
Chemical remediation	Soil washing; stabilization/solidification; chemical immobilization; soil amendments	High removal efficiency; Suitable for small to medium contamination levels	High reagent consumption; toxicity and hazard risks of chemical agents; possible soil degradation; secondary pollution risks
Biological remediation	Phytoremediation; plant-microbe combined remediation	Cost-effective for large areas; environmentally friendly; suitable for large areas; in situ application; minimal ecosystem disturbance	Slow remediation rate; dependent on plant growth and environment; low phytoextraction efficiency; unsuitable for heavily contaminated sites

**Table 3:** Selected plants with potential for Sr remediation.

Plant Species	Remediation Effect	Reference
*Amaranthus retroflexus* L., *Brassica juncea* (L.) Czern., *Phaseolus acutifolius* A. Gray	The ^90^Sr CR values for *A. retroflexus*, *B. juncea*, and *P. acutifolius* were 6.5, 8.2, and 15.2, respectively. Although *A. retroflexus* had the lowest CR, its relatively large biomass resulted in the highest total ^90^Sr accumulation. Assuming two harvests of *A. retroflexus* per year, approximately 7 years would be required to reduce ^90^Sr contamination by 50%.	[126]
*Calotropis gigantea* R.Br.	In a culture solution containing 5 × 10^3^ kBq/L ^90^Sr, *C. gigantea* removed 90% of the radionuclide within 24 h, indicating its potential for ^90^Sr phytoremediation.	[127]
*Chrysopogon zizanioides* L. Roberty.	When ^90^Sr was added alone, *C. zizanioides* achieved a cumulative removal efficiency of 94% at the end of the experiment (168 h). When ^90^Sr and ^137^Cs were added together, 91% of ^90^Sr and 59% of ^137^Cs were removed from the solution after 168 h.	[128]
*Euphorbia macroclada* Bioss., *Verbascum cheiranthifolium* Bioss., *Astragalus gummifer* L.	In soils collected from the semi-arid Keban mining area, Sr concentrations in shoots, roots, and soil were 453, 243, and 398 mg/kg for *E. macroclada*, 149, 106, and 398 mg/kg for *V. cheiranthifolium*, and 278, 223, and 469 mg/kg for *A. gummifer*, respectively. The aboveground biomass of these species may therefore be useful for decontaminating polluted soils.	[129]
*Broussonetia papyrifera* L., *Parthenocissus quinquefolia*(L.) Planch.	Among plant species collected from a uranium tailings pond in South China, *P. quinquefolia* exhibited the strongest Sr removal capacity, with a removal amount of 3920 μg.	[130]
*Arabidopsis thaliana* (L.) Heynh., *Sesbania grandiflora* (L.) Pers.	Transgenic *A. thaliana* and *S. grandiflora* showed 70% and 60% higher ^90^Sr enrichment, respectively, compared with control plants, resulting in a corresponding 50% decrease in soil ^90^Sr content.	[131]
*Triticum aestivum* L., *Avena sativa* L., *Avena nuda* L., *Hordeum vulgare* L.	At both 100 and 500 mg Sr/kg, Sr had no significant effect on shoot biomass accumulation, tillering, or maturation. Barley and naked oat accumulated the highest Sr concentrations, whereas wheat showed the lowest accumulation. Among the tested cultivars, ‘Neimengkeyimai-1’ exhibited the highest Sr concentration and appears to be a promising candidate for phytoremediation of contaminated soils.	[100]
*Sorghum bicolor* L.	Sr concentrations in roots, stems, and leaves increased linearly with the amount of Sr added to the soil (R^2^ > 0.95). At a soil Sr amendment of 400 mg/kg, the mean Sr concentrations in roots, stems, and leaves reached 68.9, 61.3, and 132.6 mg/kg (dry weight), respectively.	[132]
*Arabidopsis halleri* L.	In a hydroponic experiment with Sr(NO_3_)_2_ concentrations ranging from 0.001 to 100 mM, *A. halleri* showed a Sr transfer factor as high as 184.	[103]
*Sorghum bicolor* L.	In non-mycorrhizal soils amended with 0, 75, 725, and 975 mg/kg Sr, shoot Sr concentrations were 8.23, 92.14, 464.43, and 632.12 mg/kg, respectively. After AMF inoculation, shoot Sr concentrations increased to 21.07, 120.89, 864.07, and 1101.31 mg/kg, respectively.	[123]
*Alstonia scholaris* (L.) R. Br.	Following a 21-day Sr stress experiment using SrCl_2_·6H_2_O, Sr accumulation in *A. scholaris* seedlings ranged from 1306.4 to 8704.7 mg/kg (dry weight) under 0.3, 1.5, and 3 mM Sr treatments, with TF values ranging from 1.45 to 84.3.	[133]
*Sorghum sudanense* (Piper) Stapf.	At a soil Sr concentration of 500 mg/kg, the annual Sr removal rate of *S. sudanense* reached 23.05%. Compared with the control, microbial consortium inoculation increased Sr accumulation in *S. sudanense* by 50–400%.	[134]
*Cucumis sativus* L.	Under combined ^88^Sr + ^133^Cs treatment, whole-plant accumulation reached 2128.5 ± 219.2 μg/g (dry weight), with a maximum TF value of 2.78, indicating efficient uptake and translocation of Sr-containing radionuclide mixtures.	[108]
*Iris germanica* L., *Nephrolepis exaltata* (L.) Schott	Both *Iris germanica* and *Nephrolepis exaltata* efficiently accumulated Sr, with TF values consistently above 1 (approximately 1.2–2.2), meeting the criteria for classification as Sr hyperaccumulators.	[135]

## Data Availability

No new data were created or analyzed in this study. Data sharing is not applicable.
